# Fibrinolytic abnormalities in acute respiratory distress syndrome (ARDS) and versatility of thrombolytic drugs to treat COVID‐19

**DOI:** 10.1111/jth.14872

**Published:** 2020-06-03

**Authors:** Claire S. Whyte, Gael B. Morrow, Joanne L. Mitchell, Pratima Chowdary, Nicola J. Mutch

**Affiliations:** ^1^ Aberdeen Cardiovascular & Diabetes Centre School of Medicine Medical Sciences and Nutrition Aberdeen UK; ^2^ Radcliffe Department of Medicine University of Oxford Oxford UK; ^3^ Institute of Cardiovascular and Metabolic Sciences School of Biological Sciences University of Reading Reading UK; ^4^ Katharine Dormandy Haemophilia and Thrombosis Centre Royal Free Hospital London UK

**Keywords:** fibrin, fibrinolysis, plasminogen activator inhibitor 1, respiratory distress syndrome (adult), SARS virus, tissue plasminogen activator

## Abstract

The global pandemic of coronavirus disease 2019 (COVID‐19) is associated with the development of acute respiratory distress syndrome (ARDS), which requires ventilation in critically ill patients. The pathophysiology of ARDS results from acute inflammation within the alveolar space and prevention of normal gas exchange. The increase in proinflammatory cytokines within the lung leads to recruitment of leukocytes, further propagating the local inflammatory response. A consistent finding in ARDS is the deposition of fibrin in the air spaces and lung parenchyma. COVID‐19 patients show elevated D‐dimers and fibrinogen. Fibrin deposits are found in the lungs of patients due to the dysregulation of the coagulation and fibrinolytic systems. Tissue factor (TF) is exposed on damaged alveolar endothelial cells and on the surface of leukocytes promoting fibrin deposition, while significantly elevated levels of plasminogen activator inhibitor 1 (PAI‐1) from lung epithelium and endothelial cells create a hypofibrinolytic state. Prophylaxis treatment of COVID‐19 patients with low molecular weight heparin (LMWH) is important to limit coagulopathy. However, to degrade pre‐existing fibrin in the lung it is essential to promote local fibrinolysis. In this review, we discuss the repurposing of fibrinolytic drugs, namely tissue‐type plasminogen activator (tPA), to treat COVID‐19 associated ARDS. tPA is an approved intravenous thrombolytic treatment, and the nebulizer form has been shown to be effective in plastic bronchitis and is currently in Phase II clinical trial. Nebulizer plasminogen activators may provide a targeted approach in COVID‐19 patients to degrade fibrin and improving oxygenation in critically ill patients.

## 1 INTRODUCTION

In early December 2019 multiple cases of pneumonia of unknown etiology were reported in Wuhan, Hubei province, China.^1^, ^2^, ^3^ In January 2020 the World Health Organization declared that this was caused by a new type of coronavirus (SARS‐CoV‐2). The spread of SARS‐CoV‐2 has been exponential, resulting in a global pandemic with more than two million confirmed cases. While most people with COVID‐19 develop only mild illness, characterized by a fever and continuous cough,^2^ approximately 14% develop severe disease that requires hospitalization and oxygen support and 5% require admission to intensive care. COVID‐19 patients with respiratory distress present primarily with severe hypoxemia, yet respiratory system compliance can vary from near normal to exceptionally low.^4^ In severe cases, patients with COVID‐19 develop a type of acute respiratory distress syndrome (ARDS), sepsis, and multi‐organ failure. Older age and co‐morbidities are associated with higher mortality.^5^

**Figure 1:**
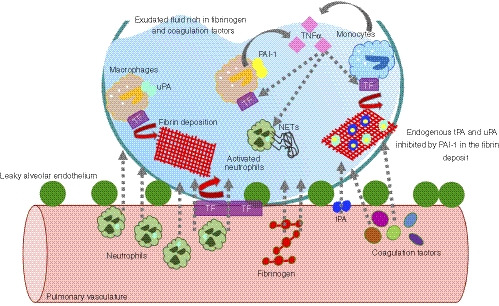
Development of fibrin deposits in the alveolar space. Development of acute respiratory distress syndrome (ARDS) is characterized by the recruitment of inflammatory leukocytes, including neutrophils, macrophages, and monocytes to the pulmonary vasculature and alveolar air space. This leads to a massive insult in the alveolar‐capillary membrane and exudation of fluid rich in cells and plasma proteins, including coagulation factors and fibrinogen. Damage to the endothelial membrane and pulmonary vasculature allows accumulation of coagulation factors within the alveoli. Tissue factor (TF) exposed on the surface of damaged endothelial cells and on the surface of macrophages and monocytes promotes fibrin formation. High levels of tissue necrosis factor β_1_ (TNF‐β_1_) activate neutrophils to form neutrophil extracellular traps (NETs) and amplify TF exposure on the surface of macrophages and monocytes. Elevated plasminogen activator inhibitor (PAI‐1) expression on the surface of monocytes and macrophages prevents degradation of fibrin deposits by inhibiting tissue plasminogen activator (tPA) and urokinase plasminogen activator (uPA)

A hallmark of ARDS is increased alveolar‐capillary permeability triggered by exudation of fluid rich in cells and plasma proteins, including albumin, fibrinogen, proinflammatory cytokines, and coagulation factors^6^, ^7^ (Figure 1). This leads to recruitment of inflammatory leukocytes, including neutrophils,^8^ alveolar macrophages,^9^ monocytes, and platelets, which propagate the local inflammatory response.^10^ Fibrin deposition in the air spaces and lung parenchyma are consistently observed with ARDS and contribute to hyaline‐membrane formation and subsequent alveolar fibrosis.^11^, ^12^, ^13^, ^14^ This promotes the development and progression of respiratory dysfunction and right heart failure.^15^ Fibrin deposition is the net result of an alteration in the balance of the coagulation and fibrinolytic pathways, and several therapeutic strategies have been explored to target the dysfunction of these systems in ARDS.^16^, ^17^, ^18^, ^19^ Recent case studies describe fibrin deposits in biopsies of lung tissue from patients with COVID‐19,^20^, ^21^ with ARDS commonly reported.^22^, ^23^ Consistent with this, large numbers of infiltrating immune cells have been found in COVID‐19 positive lung tissues, particularly monocytes and macrophages,^21^, ^23^, ^24^, ^25^ alongside the formation of fibrin,^15^, ^21^, ^25^ proteinaceous hyaline membranes, and pulmonary fibrosis.^24^, ^25^ Computed tomography (CT) scans of COVID‐19 patients’ lungs reveal characteristic ground glass opacities (GGO), indicating partial filling of the bronchoalveolar airspace with exudate.^26^, ^27^ The timing of the accidental sampling in the COVID‐19 patients with lung cancer suggests these early fibrin lung depositions present prior to clinical symptoms of pneumonia.^21^ Therefore, biomarkers to allow early identification of these changes would be highly beneficial in early diagnosis and timely treatment of COVID‐19 patients. This review will focus on the molecular mechanisms and role of inflammatory cells in underpinning fibrin deposition and persistence in the lungs of critically ill COVID‐19 patients and discuss potential therapeutic strategies to help support these patients.

## 2 THE INFLAMMATORY RESPONSE IN ARDS

Sequestration of leukocytes, particularly neutrophils, within the microvasculature of the lung is central to the development of ARDS, leading to a massive insult to the alveolar‐capillary membrane, unrestricted inflammation, and microthrombus formation (reviewed by Matthay et al^28^). Neutrophils, resident alveolar macrophages, and monocyte‐derived macrophages, as well as recruited monocytes, infiltrate the lungs, enhance lung injury, and play a key role in the pathogenesis of ARDS.^29^, ^30^, ^31^, ^32^ Release of proinflammatory cytokines, including macrophage inflammatory protein 2 (MIP‐2), interleukin 8 (IL‐8), interleukin‐6 (IL‐6), interleukin‐10 (IL‐10), and tumour necrosis factor α (TNF‐α), encourage ongoing infiltration of immune cells from the intravascular compartment to the alveolar airspaces.^33^, ^34^, ^35^ Indeed, these proinflammatory cytokines are used as biomarkers of ARDS and have been suggested to be important in progression of COVID‐19 associated ARDS.^28^

Accumulation of coagulation factors in the lungs can also drive ARDS through the activation of protease‐activated receptors (PARs), which are expressed on cells in the lungs including alveolar epithelial cells, fibroblasts, monocytes, and macrophages.^36^, ^37^ PAR signalling induced by tissue factor, coagulation factor Xa, factor VIIa, or thrombin can augment fibrosis in addition to driving fibrin generation. Fibrosis is characterized by fibroblast migration, proliferation, and deposition of collagen in the intra‐alveolar spaces. PAR‐1 can be acted upon in fibroblasts by both thrombin and factor Xa to promote their proliferation, induce production of pro‐collagen, and amplify expression of various growth factors including connective tissue growth factor (CTGF).^38^, ^39^ PAR signalling can enhance inflammation in acute lung injury (ALI) by increasing the expression of pro‐inflammatory cytokines, including IL‐6,^40^ IL‐8,^40^, ^41^, ^42^ and platelet derived growth factor.^43^

Accumulation of neutrophils in the lungs further contributes to the pathophysiology of ARDS.^28^ Neutrophils release their DNA alongside their nuclear and cytoplasmic contents into the extracellular environment during the cell death process, NETosis. These web‐like cellular extrusions, termed neutrophil extracellular traps (NETs) form a scaffold of chromatin decorated with cytoplasmic and granule proteins and histones. NETs play a role in the fight against invading pathogens. However, if not tightly regulated, NETs can contribute to the pathogenesis of non‐infectious diseases where they can exacerbate coagulation and inflammation^44^ and have recently been reported as a contributing player in the pathogenesis of ARDS and ALI where they cause further damage to the lungs.^45^, ^46^ NET production has been identified in the lungs during ARDS, where levels of NETs are greatly increased in the bronchoalveolar lavage (BAL) of both ARDS patients and mouse models of induced ALI and ARDS.^45^, ^47^, ^48^, ^49^ Increased NETs correlate with the severity of ARDS^45^, ^48^ and disease severity is reduced in mouse models when NETs are degraded using DNase1.^45^

## 3 DYSREGULATION OF COAGULATION AND FIBRINOLYSIS IN ARDS

A hypercoagulable state exists in the lungs of ARDS patients, leading to the deposition of fibrin in the intra‐alveolar space^50^ (Figure 1). Inflammation modulates coagulation by activating C‐reactive protein (CRP), thereby augmenting tissue factor exposure on monocytes and alveolar macrophages^51^, ^52^ which in turn promote thrombin generation and deposition of fibrin. Hepatic synthesis of fibrinogen, an acute phase protein, is increased 2‐ to 10‐fold in plasma during infection^53^ and local synthesis in the lung epithelium is evident during pneumonia^54^ thereby further exacerbating fibrin deposition. Fibrin deposition augments inflammation and fibrosis as well as damaging lung surfactant.^49^, ^55^, ^56^

This is coupled with a hypofibrinolytic state in the alveolar space, where fibrinolytic inhibitors have been shown to be elevated. Levels of thrombin activatable fibrinolysis inhibitor (TAFI) and protein C inhibitor were found to be significantly elevated in the bronchoalveolar fluid of patients with interstitial lung disease when compared to healthy controls.^57^ Furthermore, it has been reported that α2‐macrogloblin levels are increased in obstructive lung disease, which may correlate with the increase in plasminogen observed in the BAL of ARDS patients.^58^, ^59^ However, the principal fibrinolytic inhibitor described in the pathogenesis of ARDS is plasminogen activator inhibitor 1 (PAI‐1), which is known to be elevated in severe acute respiratory syndrome coronavirus (SARS‐CoV) and ALI.^11^, ^60^

In ARDS, CRP promotes local release of PAI‐1 from endothelial cells.^61^, ^62^ Additionally, infiltration of platelets, the major circulating pool of PAI‐1, may result in local release. We have recently shown that a significant amount of this active PAI‐1 remains associated with the stimulated platelet membrane.^63^, ^64^ Elevated levels of PAI‐1 in ARDS depresses urokinase (uPA) activity in the bronchoalveolar fluid.^11^ Attenuation of the plasminogen activation system leads to abnormal turnover of fibrin in the alveolar space. Plasma PAI‐1 levels have been reported as an independent risk factor for poor prognosis and mortality in ALI.^59^, ^61^, ^62^, ^65^, ^66^, ^67^, ^68^ Prabhakaran et al^61^ reported a significant increase in PAI‐1 antigen and activity in plasma and the edema fluid in ALI, with evidence of significant pulmonary production.^61^ A clear role for PAI‐1 as a prognostic marker in ARDS was confirmed by a prospective observational study which demonstrated 5‐fold higher levels in patients who progressed to ARDS than those with uncomplicated aspiration pneumonitis (2687 versus 587 ng/mL, respectively).^67^

Importantly, a hypofibrinolytic state and increased PAI‐1 was observed in the SARS‐CoV epidemic in 2002 and 2003.^60^ Gralinski et al used a non‐biased systems biology approach to study the dysfunctional fibrinolytic pathway in an infection model of SARS‐CoV.^60^ Fibrin persistence was mediated by over‐expression of PAI‐1, which overcomes local uPA and tissue‐type plasminogen activator (tPA).^60^ SARS‐CoV infected cells contain high levels of TGF‐β1, which in turn stimulates expression of extracellular matrix protease inhibitors, including PAI‐1,^68^ which has been specifically linked to ARDS induced by SARS‐CoV.^69^ These studies illustrate a clear role for PAI‐1 in the etiology of ARDS and suggest it is a key protein contributing to abnormal turnover of fibrin in the alveolar space.

Plasma PAI‐1 has been reported as a potential biomarker for predicting disease progression in ALI to ARDS, with one study concluding that PAI‐1 antigen > 640 ng/mL was a 100% positive predictor for mortality.^61^ Similar pathology of fibrin depositions in the lungs has been identified in COVID‐19,^21^, ^25^ suggesting PAI‐1 may be a useful prognostic marker for patients at risk of developing ARDS and thus requiring critical care and ventilation.

## 4 THERAPEUTIC OPTIONS FOR ARDS IN COVID‐19 PATIENTS

A common finding with COVID‐19 patients requiring hospitalization is increased levels of D‐dimers and fibrin degradation products (FDP) which are associated with a higher risk of mortality.^70^ Prothrombin time and activated partial thromboplastin time show a mild elongation.^70^ Coupled with the fact critically ill COVID‐19 patients will be immobilized, there is an increased risk of hospital‐associated venous thromboembolism (VTE).^71^ These findings have led to a recent recommendation for prophylactic anticoagulant therapy with low molecular weight heparin (LMWH) for patients hospitalized with COVID‐19, without contraindications, to limit the extent of the coagulopathy.^72^, ^73^ Heparin treatment (both unfractionated and LMWH) reduces inflammatory biomarkers,^74^ and therefore may be beneficial in reducing the inflammatory state in COVID‐19. Disseminated intravascular coagulation (DIC) is often observed in patients with ARDS where fibrin and microthrombi are detected in the lungs^12^ and BAL.^59^ Consistent with this, numerous patients infected during the SARS‐CoV epidemic in 2002‐2003 displayed DIC^75^ and elevated levels of fibrinogen^76^ and D‐dimers.^77^

Anticoagulant therapy is essential to limit ongoing fibrin deposition and microthrombi formation in ARDS and treat the systemic prothrombotic complications in these patients. However, LMWH will be ineffective in clearing fibrin clusters deposited in the alveolar space. There is therefore a requirement to readdress the balance of fibrinolysis in the lung, either by enhancing plasminogen activation or downregulating fibrinolytic inhibitors. The significant increase in PAI‐1 in ARDS and ALI curtails local uPA, but also tPA, activity.^11^, ^17^, ^78^, ^79^ In a pig model of trauma, administration of tPA or uPA prevented development of ARDS, with animals displaying normal PaO_2_.^80^ A phase 1 clinical trial revealed a significant improvement in PaO_2_ at 24 hours in 19 out of 20 patients with severe ARDS secondary to trauma or sepsis following administration of uPA or streptokinase.^81^, ^82^ These patients had a PaO_2_ of <60 mm Hg, usually considered fatal, which increased to 231.5 mm Hg following thrombolytic therapy with an overall 30% survival rate and no incidence of bleeding.^82^ The use of tPA to treat ARDS in COVID‐19 patients has recently been proposed by Moore et al.^15^ An initial case report from three patients from the current SARS‐CoV‐2 pandemic demonstrates a transient improvement in P/F ratio in two cases and sustained 50% improvement in one case following administration of a 25 mg bolus of intravenous tPA followed by a further 25 mg infusion.^83^ The authors suggest that there is a precedent for increasing the dose of the bolus of tPA while maintaining heparin infusion, as the anticoagulant is effective against sub‐massive pulmonary embolism.^83^, ^84^ In addition to readdressing the fibrinolytic balance, administration of tPA to ARDS patients may confer anti‐inflammatory effects, as it has been shown to suppress neutrophil activation in a rat model of ALI induced by IL‐1α.^85^

A major consideration in anticoagulant or thrombolytic therapy is the undesirable complication of bleeding. In respiratory medicine, treatments are often delivered as aerosolized protein therapeutics as diffusion of proteins from the blood to the lungs can be limited.^86^ Interestingly, nebulized anticoagulant therapy with antithrombin or heparin has been shown to reduce lung injury without an increase in systemic bleeding in animal models^87^, ^88^, ^89^ and ALI patients.^90^ However, as discussed, heparin will prevent further fibrin deposition but will be ineffective in the removal of pre‐existing fibrin. A recent publication compared the efficacy of the nebulized form of the plasminogen activator, streptokinase, and nebulized heparin in the treatment of ARDS.^91^ The primary outcome in this trial was the change in PaO_2_/FIO_2_ ratio, which was significantly higher in the streptokinase group from day 1 to day 8, compared to the heparin and standard‐of‐care groups. Importantly, intensive care unit (ICU) mortality was significantly lower in streptokinase patients compared to other groups.^91^ A 1999 case report^92^ describes a young woman with ARDS who was resistant to conventional therapeutics and was treated with nebulized and intravenous tPA, followed by continuous treatment with nebulized unfractionated heparin. The patient stabilized following fibrinolytic treatment and demonstrated a significant enhancement in pulmonary gas exchange.

**Figure 2:**
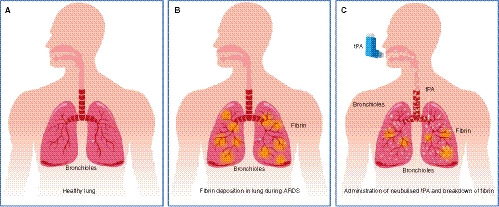
Fibrin deposition in the lungs during acute respiratory distress syndrome (ARDS) and breakdown by nebulised tissue plasminogen activator (tPA). A, Normal healthy lung with no detectable fibrin deposits. B, During development of ARDS the equilibrium between coagulation and fibrinolysis is disrupted resulting in fibrin deposits in the lung parenchyma and fibrin‐platelet microthrombi in the pulmonary vasculature. This promotes respiratory dysfunction and can lead to a requirement for respiratory support. C, Administration of nebulized tPA will target the bronchioalveolar space tipping the balance of plasminogen activation in favor of fibrinolysis allowing clearance of fibrin from the lung parenchyma thereby improving respiratory function and oxygenation in COVID‐19 patients

Plastic bronchitis is a condition that can develop from several respiratory disorders, resulting in casts of compacted mucous that have been shown to contain fibrin.^93^ Plastic bronchitis is primarily observed in children and has been described in cases of influenza A (H1N1)^94^, ^95^ and human bocavirus.^96^ Nebulized tPA has been shown to be effective in preventing recurrent cast formation in plastic bronchitis.^93^ Reports thus far are from single case studies; however, there is an ongoing phase II clinical trial of nebulized tPA (PLATyPuS; alteplase, NCT02315898) for treatment of plastic bronchitis. These data clearly indicate that use of nebulized fibrinolytics could allow a more targeted approach to correct the hemostatic imbalance that results in fibrin deposition, while limiting the risk of systemic activation of fibrinolysis that may trigger unwanted bleeding (Figure 2). Inhaled tPA is absorbed into the vasculature thus increasing fibrinolytic capacity in the plasma^97^ and the potential to lyse the microthrombi observed in COVID‐19 patients. However, it is conceivable that intravenous infusions of tPA may be necessary to disperse larger thrombi in the circulation. A potential caveat of a nebulizer formulation is that aerosolized proteins are susceptible to degradation so the formulation and excipient used must be considered.^86^ However, in the case of tPA, an extreme advantage is that a formulation of nebulized alteplase has been developed and is currently being tested in a Phase II clinical trial.^86^

## 5 CONCLUDING REMARKS

The COVID‐19 global pandemic has necessitated a demand for novel therapeutics to limit the complications of ARDS and/or reduce the burden on ventilatory support in intensive care units. The indication that fibrin deposits occur prior to symptoms^21^ of the disease suggests that targeting the fibrinolytic system to promote fibrin resolution could limit severity and improve pulmonary function. Given the urgent time scale of the clinical requirement, repurposing of existing therapies, such as nebulized tPA, to promote fibrin dissolution in the lung and improve oxygenation is a pragmatic approach in addressing the ARDS complications associated with COVID‐19.

## CONFLICTS OF INTEREST

CSW, GBM, JLM, and NJM have no conflicts of interest to declare. PC received research funding from CSL Behring, Pfizer, NovoNordisk, SOBI, and Bayer; and is on the advisory boards and speaker bureau for Baxalta, Bayer, Biogen, CSL Behring, Chugai, Pfizer, Freeline, NovoNordisk, Roche, SOBI, and Shire.

## AUTHOR CONTRIBUTIONS

NJM and PC conceived the idea. CSW, GBM, JLM, and NJM all wrote and edited the manuscript.
